# Complete Genome Sequence of Campylobacter jejuni YH001 from Beef Liver, Which Contains a Novel Plasmid

**DOI:** 10.1128/genomeA.01492-14

**Published:** 2015-02-05

**Authors:** Yiping He, Xianghe Yan, Sue Reed, Yanping Xie, Chin-Yi Chen, Peter Irwin

**Affiliations:** Molecular Characterization of Foodborne Pathogens Research Unit, U.S. Department of Agriculture, Agricultural Research Service, Eastern Regional Research Center (USDA-ARS-ERRC), Wyndmoor, Pennsylvania, USA

## Abstract

Campylobacter jejuni, commonly found in poultry and meat products, causes gastroenteritis in humans. Here, we report the complete genome sequence of a C. jejuni strain, YH001, isolated from retail beef liver. The genome is 1,712,361 bp and has a 30.5% G+C content and two plasmids of 46.5 kb and 4.4 kb.

## GENOME ANNOUNCEMENT

Campylobacter jejuni is a Gram-negative microaerophilic bacterium that colonizes the intestines of most warm-blooded hosts, including all food-producing animals and humans. The processing of poultry and other food-producing animals often results in pathogen contamination to consumable food products, leading to human infection. The prevalence of Campylobacter in retail meat products can be as high as 70% (1, 2). Each year, >0.8 million cases of Campylobacter illness are reported in the United States, with approximately 8,463 hospitalizations and 76 deaths (3). Additionally, the misuse of antibiotics in food-producing animals and environments has resulted in an increasing occurrence of antibiotic resistance in Campylobacter, which subsequently affects the efficacy of antibiotic treatment (4). Studies have shown that the colonization, adherence, and invasion of host epithelial cells and the induction of cell apoptosis are important virulence factors in Campylobacter infection, but the mechanism of pathogenesis is still poorly understood (5). The complete genome sequence and annotation of a new C. jejuni isolate from beef liver reported here will help identify the potential genetic determinants of virulence and antibiotic resistance in the pathogen.

C. jejuni YH001 was isolated from a beef liver purchased from a local supermarket, using a passive filtration method (6, 7). The genus and species information of the strain was first confirmed by 16S rRNA gene sequencing and multiplex real-time PCR (8, 9). Genomic DNA was extracted using the DNeasy blood and tissue kit (Qiagen) and subjected to sequencing using an Ion Torrent PGM sequencer and Ion 318 Chip (Life Technologies), following the preparation of a 400-bp fragment library and emulsion PCR amplification. The average read length is >300 nucleotides, with 350,000 to 450,000 reads per genome. The raw sequence reads were trimmed and processed using CLC Genomics Workbench 7.0 (Qiagen) and Sequencher 5.2.2 (Gene Codes) to yield *de novo* assemblies. The contigs were aligned with other Campylobacter genomes for gap-filling using long PCR and Sanger sequencing. The final assembly and potential misassemblies were validated by Sanger sequencing. All putative frameshifts were manually curated based on the coverage and quality of the given nucleotides via genome mapping. Automated gene and pseudogene prediction and annotation were performed using the PROKKA 1.10 software (10).

C. jejuni YH001 has a circular chromosome of 1,712,361 bp (30.5% G+C content), encoding 1,869 proteins. In addition, the strain carries two plasmids of 46,527 bp and 4,356 bp. Several structurally distinct and highly divergent type III secretory proteins were found in the large plasmid, which may represent environmental adaptation. The main virulence factor encoded by the cytolethal distending toxin gene cluster *cdtABC* is present in this strain. The Campylobacter multidrug efflux pump (CmeABC) and DNA gyrase, contributing to antibiotic resistance, were also found. A whole-genome comparison revealed that C. jejuni YH001 isolated from beef liver was phylogenetically distinct from most of the other C. jejuni strains in GenBank, particularly in the large plasmid containing C. jejuni integrated elements (CJIEs).

### Nucleotide sequence accession number.

The sequence has been deposited in GenBank under the accession no. CP010058.
